# Invasive Aspergillosis Associated with a Foreign Body

**DOI:** 10.1155/2015/875168

**Published:** 2015-05-10

**Authors:** Akifuddin Syed, Prashanth Panta, Imran Shahid, David H. Felix

**Affiliations:** ^1^Princess DurruShehvar Children's and General Hospital, Purani Haveli, Hyderabad, Telangana 500002, India; ^2^Department of Oral Medicine and Radiology, MNR Dental College and Hospital, Narsapur Road, Sangareddy, Medak District, Telangana 502294, India; ^3^NHS Education for Scotland, Westport 102, WestPort, Edinburgh EH3 9DN, UK

## Abstract

Invasive aspergillosis is a serious complication in immunocompromised individuals. It is associated with a high mortality rate, which demands a combined approach involving radical surgery and antifungal therapy. Here, we describe a patient who presented with nonspecific fever, refractory to antimicrobial agents. Though it primarily involved the nasal cavity and sinuses, it perforated into the oral cavity causing palatal changes. Surprisingly, a foreign body was found in the involved tissues that might have acted as a nidus of infection. A sufficient dose (3 mg/kg/day) of liposomal amphotericin B was initiated soon after a thorough debridement procedure and the patient survived.

## 1. Introduction

Aspergillosis is an infection caused by a group of soil dwelling fungal organisms. About 837* Aspergillus* species are now known of which only a few are implicated in human disease [1]. The conidia of these organisms germinate into hyphae filaments and result in disease. B-glucans present in the cell walls of these hyphae along with other ligands can precipitate a severe inflammatory response.* Aspergillus* species also release enzymes (proteases, peptidases, and phospholipases) and toxins (14 kDa diffusible substance from conidia, fumigaclavin C, aurasperon C, gliotoxin, helvolic acid, fumagillin, hemolysin, and ribotoxin) which mediate invasion and tissue necrosis [2]. The pathogenicity of aspergillosis is dependent on two factors, the involved fungal strain and the immune status of the host.

Patients with hematological malignancies, HIV/AIDS, neutropenia, and multiple myeloma and those who received bone marrow transplantation are at risk of invasive aspergillosis [3]. There is some male predilection and the most frequent sites include lungs, liver, spleen, bone, meninges, sinuses, and oral cavity. Primary intraoral aspergillosis is rare and it usually involves gingiva, palate, and rarely tongue. However,* Aspergillus* infection might spread from the maxillary sinus into the palate. Aspergillosis can broadly be divided into invasive and noninvasive forms. The most frequently isolated strains in invasive aspergillosis are* Aspergillus fumigatus* and* Aspergillus flavus* [4]. In the present case, the culprit organism was* Aspergillus niger*, a species that is less pathogenic to humans, suggesting the role of nonfumigatus organisms in invasive aspergillosis.

## 2. Case Report

**Figure 1:**
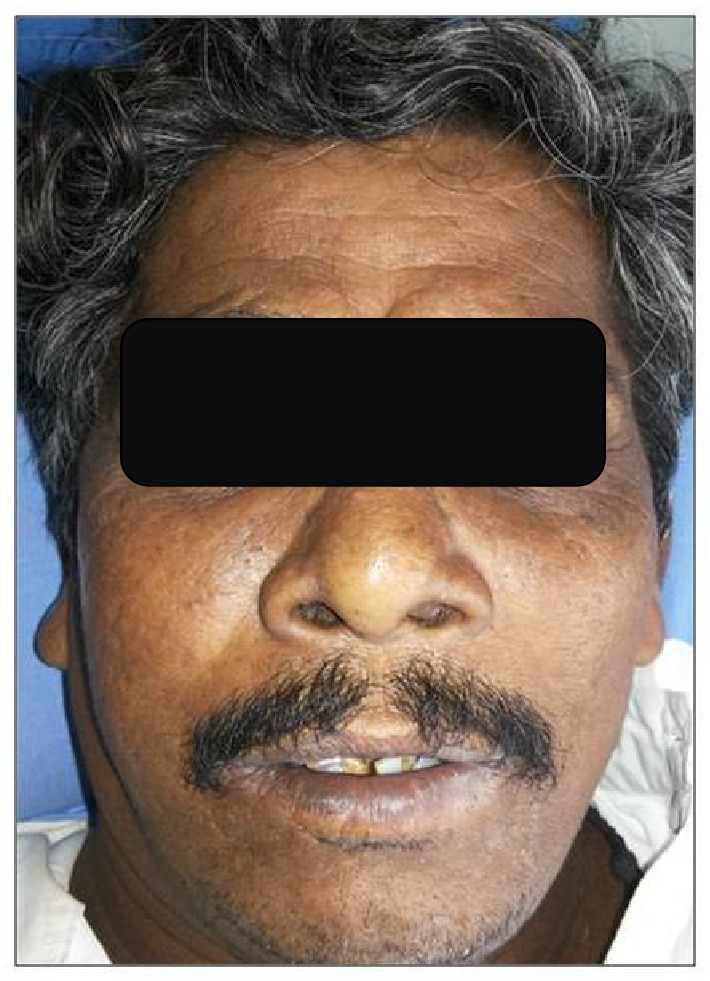
Diffuse facial swelling and mild bilateral obliteration of the nasolabial folds.

**Figure 2:**
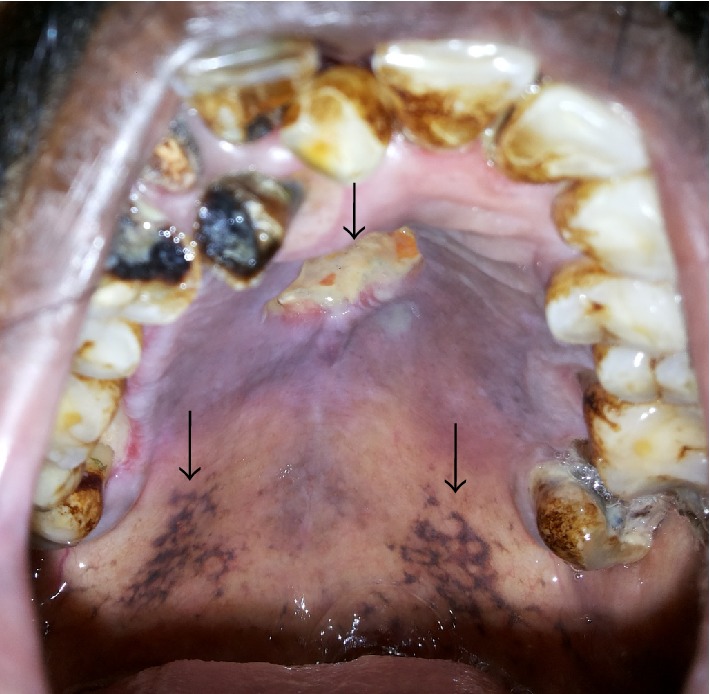
Diffuse oedematous swelling of the palatal mucosa with focal ulceration. Note violaceous discolouration with black pigmented areas over soft palate.

A 51-year-old male farmer initially reported to a general physician with the chief complaint of fever and malaise. Routine blood investigations revealed leukocytosis (15,200/mm^3^), but the source of sepsis could not be identified and his condition was refractory to empirical antimicrobial therapy. His medical history included diabetes diagnosed ten years earlier; he was a heavy smoker for the last 20 years. On day 5, the patient complained of acute pain from the upper left central incisor region, so, he was reviewed by a maxillofacial surgeon. Upon extraoral examination, a diffuse swelling (Figure 1) was found on the left and right malar region; there was no cervical lymphadenopathy. Intraorally, the palatal gingiva showed a yellow ulcerated area (3 × 1 cm) in the anterior hard palate and many blackish areas near the soft palate region (Figure 2). The palatal mucosa was grayish-violet, blanched, and oedematous and all his upper teeth showed mobility ranging from grade I to II.

**Figure 3:**
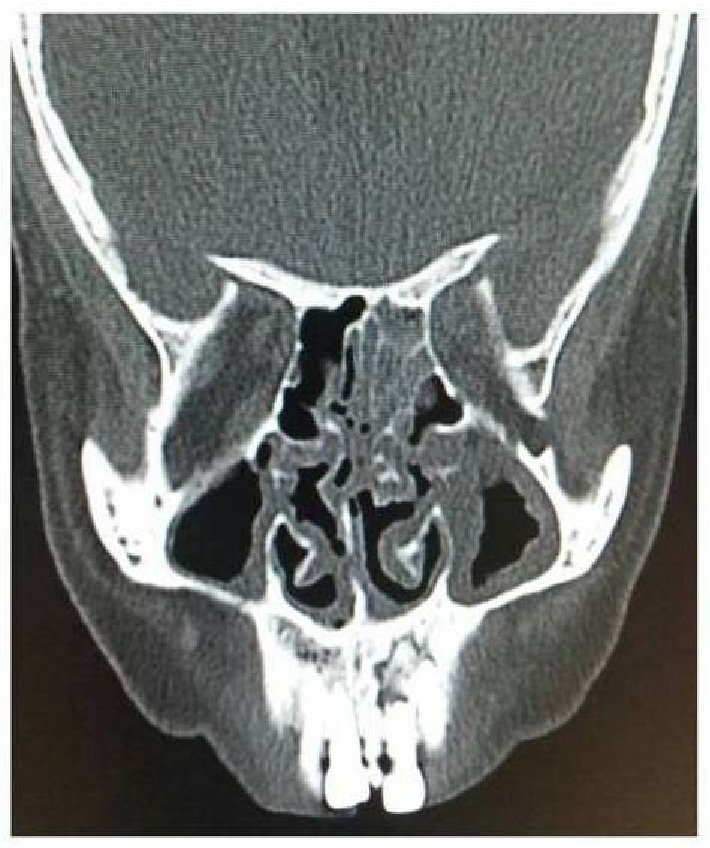
CT scan images shows thickening of mucosa and opacification of the sinuses.

**Figure 4:**
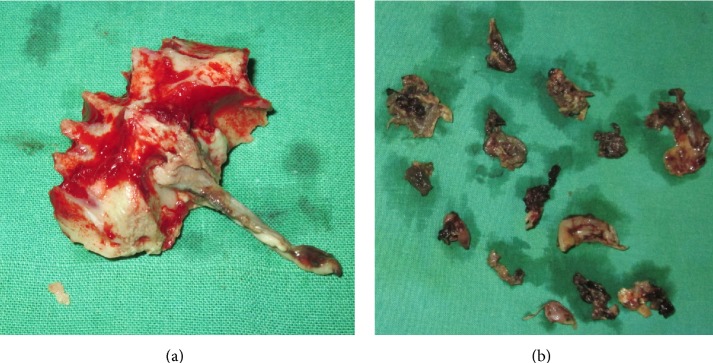
(a) Excised maxilla. (b) Black muddy areas were found in the nasal cavity, alveolar process, and the sinus region.

**Figure 5:**
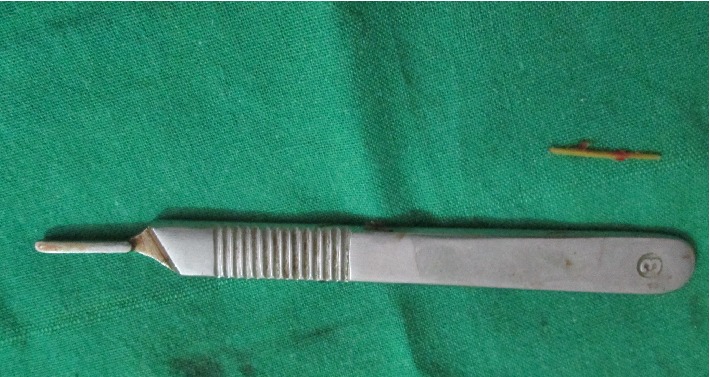
Image shows a foreign body that corresponded to a plant leaf.

**Figure 6:**
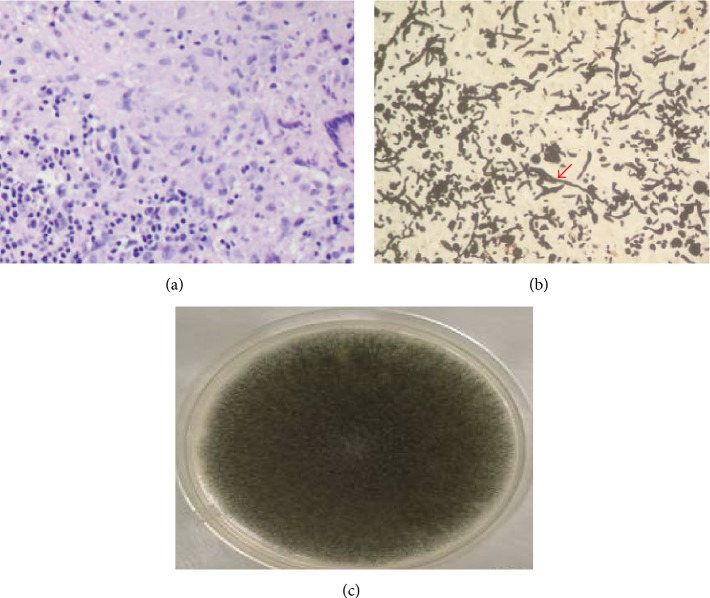
Microbiological features suggested aspergillosis caused by* niger*.

A chairside KOH test from the palatal ulcer showed features suggestive of a fungal aetiology, but the type was in question. A provisional diagnosis of fungal osteomyelitis was proposed. Following this, a decision was taken, to perform a debridement procedure, aiming at improving the patient's condition. A preoperative CT scan showed features, suggestive of erosive sinusitis with nasal involvement (Figure 3). Maxillectomy (Figure 4(a)) was performed, sparing the tuberosity region alone. Several muddy areas were found in the nasal cavity, maxillary sinus, and the alveolar process (Figure 4(b)). In the middle of these muddy areas was a foreign body measuring approximately 2.5 cm in its maximum dimension (Figure 5). Further analysis of the foreign body showed this to be a fragment of a plant leaf. Hematoxylin and eosin stained section (Figure 6(a)) showed fungal elements, and Gomori methanamine silver stained section (Figure 6(b)) showed 45° branching hyphae (pointed by a red arrow), a feature characteristic of* Aspergillus niger*. The histopathological features suggested aspergillosis, caused by niger.* Aspergillus niger* was also grown on Sabaraud's dextrose agar and the colonies were blackish in color (Figure 6(c)). The patient was started on intravenous liposomal amphotericin B (3 mg/kg/day for 5 weeks). He responded very well to this drug and had complete recovery. One month postoperatively, he was provided with an obturator to close off the palatal defect.

## 3. Discussion

Invasive aspergillosis is a rapidly spreading infection and is more likely to occur in patients with an underlying immune deficiency.* Aspergillus fumigates* (80–90%) is the most common cause of invasive aspergillosis followed by* Aspergillus flavus* (5–10%) and* Aspergillus niger* (1–5%) [5].

The first case of aspergillosis of the paranasal sinus was described by Katzenstein in 1983. Sinonasal aspergillosis can be invasive (chronic indolent and invasive fulminant sinusitis) or noninvasive (aspergilloma and allergic* Aspergillus* sinusitis) [6]. The present case may represent a chronic indolent fungal sinusitis progressing into an invasive fulminant fungal sinusitis. Chronic indolent fungal sinusitis occurs in patients with altered immune responsiveness (e.g., diabetes mellitus) [7]. The invasive form can extend into the oral cavity causing palatal perforation. In such cases, gray-violaceous gingival changes are found that soon transform into necrotic lesions. Proptosis is another important feature of paranasal and orbital aspergillosis.

In the past, foreign bodies were shown as an etiological factor for aspergillosis [8, 9]. Burnham and Bridle reported a case of maxillary sinus aspergilloma which was triggered due to extrusion of an amalgam filling [10]. Liston and Walters presented a case of* Aspergillus* sinusitis where six gutta percha points served as etiological agents after being accidentally introduced into the maxillary sinus [11]. In the present case, a foreign body was discovered in the nasal cavity that corresponded to a “plant leaf.” It was found in the middle of necrotic tissues and may have served as a source of contamination. The epicenter of tissue destruction was predominantly in the paranasal sinus region and the nasal cavity. No actual palatal perforation occurred although there was some ulceration. The large size of the foreign body (2.5 cm) and the absence of palatal perforation clearly point at the nasal route, as the probable route of entry. The patient may have accidentally inhaled the foreign body during agricultural activities.

*Aspergillus* sps. are found in soil, stored grains, litter, and decaying plant leaves [3]. They exist in spore form on dried leaves and derive the necessary nutrients from it. It is possible that spores of* Aspergillus* sp. on the foreign body/plant leaf settled in his nasal cavity. Due to his already immunocompromised and ketoacidic state, the spores may have attained full growth potential.

*Aspergillus niger/black aspergilli* is a group of black-shaded spore producing organisms that can survive even in hostile environments reflected in their ability to withstand extremes of temperature and humidity [12]. Their ability to withstand extreme temperatures and humidity may be the reason for their colonization in nasal and paranasal areas, a relatively warm and moist environment. A foreign body further complicates the scenario, favouring fungal growth.

Clinically, aspergillosis can mimic mucormycosis, an entity which is relatively common in diabetics. A differentiation between the two is possible microscopically. Special stains such as periodic acid Schiff and methenamine silver along with fungal culture can confirm the diagnosis. Recently, galactomannan assay, B-glucan assays, and polymerase chain reaction have shown high sensitivity and specificity in the detection of invasive aspergillosis [3]. Differential diagnosis of single palatal ulcer such as the one presented in this case includes oral tuberculosis, necrotizing sialometaplasia, syphilis, malignant ulcer, and deep fungal infection. Such ulcers must caution the clinician to consider the possibility of fungal infection, especially when the host is immunecompromised. In this case, there was sinus opacification with erosion of the medial and lateral walls of maxillary sinus, bony nasal septum, and alveolar process of the maxilla. There was severe mucosal thickening of the turbinates bilaterally and also deviation of the nasal septum. Radiological findings of invasive sinus disease include soft tissue mass, opacification, necrosis of multiple sinuses, and bone destruction [13].

Though amphotericin has some toxic side effects, most of the patients usually tolerate this drug well. Emerging antifungal agents such as voriconazole, echinocandins, or caspofungin alone or in combination with amphotericin/voriconazole have shown good clinical results [3]. Future studies should focus on dosage standardization, which is not well established.

## 4. Conclusion

This paper reports a case of invasive aspergillosis in a male farmer, caused by* Aspergillus niger*, a species that is believed to be less pathogenic than other strains. Invasive aspergillosis involving nasal cavity and sinuses can be expected in farmers and horticulture workers. Environmental factors may play some role in this disease, so taking a thorough occupational history is crucial for diagnosis. Single palatal ulcers along with gray-violet discolouration of the gingiva may be early indicators of invasive sinonasal aspergillosis. It is important that invasive aspergillosis is considered in patients with an underlying immune deficiency such as diabetes and in patients who present with nonspecific fever, refractory to antimicrobials. In advanced cases, a combination of radical surgical debridement and antifungal therapy should be considered. Systemic dissemination and the high mortality associated with this condition should persuade researchers to work on newer methods of identification and treatment.
